# Genetic characterization of Erve virus, a European Nairovirus distantly related to Crimean-Congo hemorrhagic fever virus

**DOI:** 10.1007/s11262-012-0796-8

**Published:** 2012-08-03

**Authors:** Meik Dilcher, Andrea Koch, Lekbira Hasib, Gerhard Dobler, Frank T. Hufert, Manfred Weidmann

**Affiliations:** 1 Department of Virology, University Medical Center Göttingen, Kreuzbergring 57, 37075 Göttingen, Germany; 2 Department of Virology and Rickettsiology, Institute of Microbiology of the Armed Forces, Neuherbergstrasse 1, 80937 Munich, Germany

**Keywords:** Erve virus, Bunyavirus, Nairovirus, Thiafora group

## Abstract

Erve virus (ERVEV) is a European Nairovirus that is suspected to cause severe headache (thunderclap headache) and intracerebral hemorrhage. The mode of transmission to humans (ticks or mosquitoes) is still unknown. Currently, no standardized testing method for ERVEV exists and only a small partial sequence of the polymerase gene is available. Here, we present the first complete genome sequence of ERVEV S, M, and L segments. Phylogenetic comparison of the amino acid sequence of the L-protein (RNA-dependent RNA polymerase) revealed only 48 % homology to available L-protein sequences of other Nairoviruses like Crimean-Congo hemorrhagic fever virus, Nairobi sheep disease virus, Hazara virus, Kupe virus, and Dugbe virus. Among themselves, these Nairoviruses show 62–89 % homology in the L-protein sequences. Therefore, ERVEV seems to be only distantly related to other Nairoviruses. The new sequence data can be used for the development of diagnostic methods and the identification of the natural vector.

## Introduction

Erve virus (ERVEV) was isolated in 1982 from tissues of a white-toothed shrew (*Crocidura russula*) collected in the Erve River Valley in northwestern France after intracerebral inoculation of young mice, which showed signs of acute encephalitis, edema, capillary congestion, cuffing, and disseminated necrosis of neurons [5]. An association with intracerebral bleeding or subarachnoid hemorrhage was assumed [24]. Ultrastructural and virological studies marked the virus a member of the *Nairovirus* genus of the *Bunyaviridae* family [5, 27]. Zeller et al. [27] showed via IFA and CF tests that ERVEV is related to Thiafora virus (TFAV), a Bunyavirus isolated 1971 from *Crocidura sp*. in Senegal. Both viruses reacted in IFA with a polyvalent serum that contained antibodies to different Nairoviruses like Crimean-Congo hemorrhagic fever virus (CCHFV), Hazara virus (HAZV), Ganjam virus (GANV) a strain of Nairobi sheep disease virus (NSDV) found in India, Dugbe virus (DUGV), and the so far unassigned Bhanja virus (BHAV), but they did not react with antibodies to either of these viruses used to prepare the polyvalent serum [27]. ERVEV was detected in France, the Netherlands, Czech Republic, and Germany [4]. A natural focus of ERVEV in the Erve River Valley is indicated by a seroprevalence in healthy blood donors of 11.4 % in contrast to 2.7 % outside of that region [5]. Seropositivity was also traced in rodents, insectivores, wild boars, red deer, sheep, and seabirds [5]. Nairoviruses are predominantly vectored by ticks, but coincidental ERVEV and *Borrelia burgdorferi* infections have not been detected, indicating that it is rather unlikely that ERVEV is transmitted by *Ixodes* or *Dermacentor* ticks [26]. Other possible vectors for ERVEV could be mosquitoes or sandflies.

A study by Treib et al. [24] implicated ERVE virus in “thunderclap headache” patients as 13.9 % of patients with this sudden excruciating headache exhibited antibodies for ERVEV. So far, only a 442-bp partial fragment of the ERVEV L segment coding for the RNA-dependent RNA polymerase (RdRP) was determined and phylogenetic comparison showed that ERVEV is a very distinct Nairovirus [11]. The genus Nairovirus consists of at least 34 strains/serotypes that have been divided into seven species/serogroups [17, 19]. The type species is Dugbe virus. Their genomes consist of 3 segments of -ssRNA: large (L), medium (M), and small (S). The L segment encodes the RdRP, the M segment the glycoprotein precursor (M-protein) for glycoproteins Gn and Gc, and the S segment the nucleocapsid protein (N). In addition, an M segment-encoded non-structural protein, NS_M_, was recently identified in CCHFV [1]. Full-length sequence data are available for CCHFV, HAZV, DUGV, and NSDV, as well as Kupe virus (KUPV) which is closely related to DUGV. CCHFV is currently the best-characterized member and the most pathogenic Nairovirus with a high average case fatality rate of 30 % (range 10–50 %) [8, 25]. It is widely distributed in Africa, the Balkans, the Middle East, Russia, and Central Asia [6, 9] and can cause diseases ranging from mild flu-like symptoms to severe hemorrhagic fever. Here, we present the first complete genome sequence and phylogenetic analysis of ERVEV, a putative human-pathogenic member of the Thiafora serogroup, which seems to represent the most distantly related Nairovirus.

## Materials and methods

### Erve virus culture and RNA extraction

ERVEV was passaged 5 times in Vero E6 cells maintained in DMEM medium supplemented with 2 % fetal bovine serum, 2 mM Glutamine, 10 mM Penicillin, and 10 mM Streptomycin in a 175-cm^2^ tissue culture flask. Culture supernatant of infected cells was collected when the cells showed 90–100 % CPE (14 dpi), centrifuged at 700×*g* (2,000 rpm) for 10 min then at 2,800×*g* (4,000 rpm) for 5 min and filtered through a 0.2-μm sterile filter. To enrich for viral particles, 20 ml supernatant was mixed with 1.48 ml 5 M NaCl and 10.8 ml 30 % PEG8000 in NTE (10 mM Tris, pH6.5; 1 mM EDTA; 100 mM NaCl), incubated on a shaker for 30 min at 4 °C, and subsequently centrifuged at 6,000 rpm for 60 min and 4 °C. The virus pellet was resuspended in 500 μl PBS. RNA extraction was performed as described [7].

### Pyrosequencing

To be able to cover the 3′ terminal parts of the segments, 500 ng self-complementary FLAC adapters were ligated to 500 ng purified viral ssRNA as described for dsRNA Orbiviruses [7, 13, 20]. To achieve coverage of the 5′ terminal parts, a 5′-RACE RNA adapter was ligated to the viral RNA after the removal of two phosphate groups via RNA 5′-Polyphosphatase treatment as described in the FirstChoice RLM-RACE Kit from Ambion. To get rid of unligated adapters, we included a purification step via the CleanAll DNA/RNA Clean-Up and Concentration-Kit (Norgen Biotek). The concentration of the adapter-ligated ssRNA was determined via Nanodrop and Qant-iT RiboGreen Assay (Invitrogen). 60 ng adapter-ligated viral RNA was subsequently amplified and converted to dsDNA using the TransPlex Whole Transciptome Amplification kit (WTA2) from Sigma-Aldrich. After purification via the QIAquick PCR Purification kit (Qiagen), an additional size exclusion step via Ampure-XP beads (Agencourt) in a ratio of 1 vol RNA to 0.7 vol Ampure-XP beads was used to remove fragments shorter than 350 bp.

300 ng of the whole genome amplified dsDNA was used for Titanium Shotgun Rapid Library Preparation and pyrosequencing as described in the FLX Titanium Protocol (Roche) and in [16], but omitting the DNA fragmentation by nebulization step. In the RL adapter ligation step, RL-MID adapters were used to allow pooling of several samples.

### Bioinformatics

#### Assembly

Assembly of the sequenced ERVEV genome segments was done by means of the Genome Sequencer FLX System Software Package version 2.3 (GS De Novo Assembler, GS Reference Mapper) in combination with the commercially available SeqMan Pro Software version 9.1.0 (DNASTAR, Lasergene).

#### General genome comparison

**Fig. 1:**
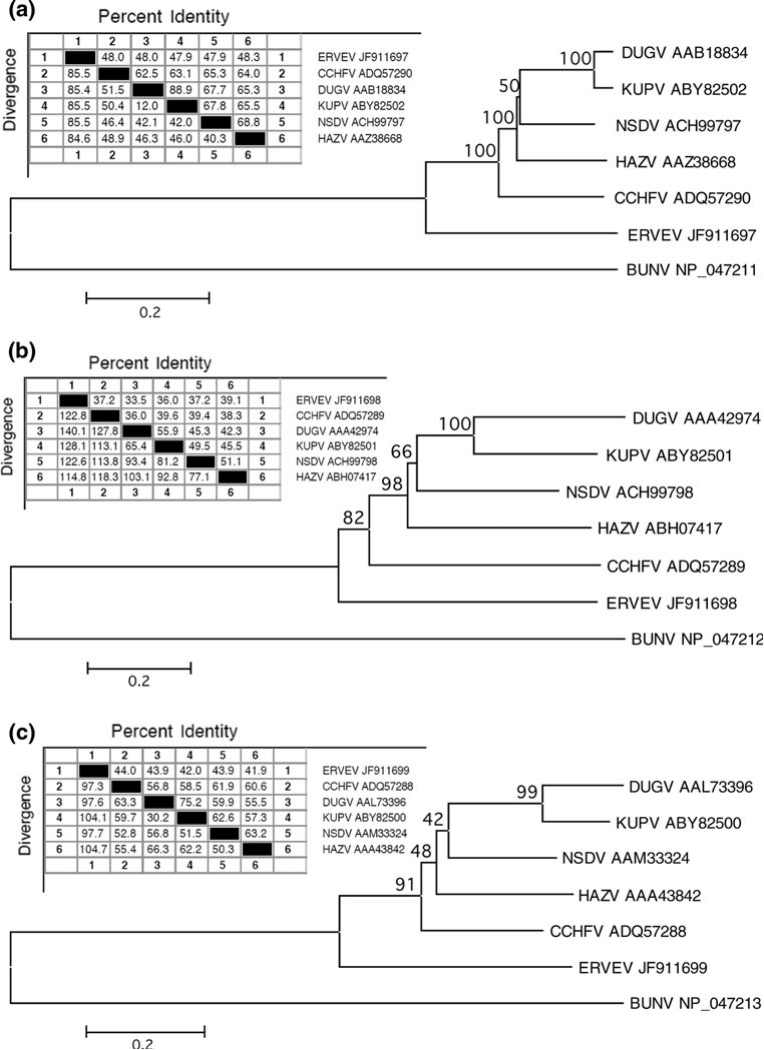
Sequence pair distances and neighbor-joining phylogenetic trees of the **a** RdRP protein sequences **b** Glycoprotein precursor protein sequences, and **c** Nucleocapsid protein sequences of different Nairovirus species rooted to the corresponding protein sequences of the Orthobunyavirus Bunyamwera virus (BUNV) by means of MegAlign version 9.1.0 (DNASTAR, Lasergene) and the MEGA4 software [21] by the ClustalW multiple alignment algorithm and a 1,000 fold bootstrap approach. Bootstrap values are given in percent. GenBank accession numbers of the used Nairovirus proteins are indicated

Additional Nairovirus genomes were downloaded from GenBank [2] and Swiss-Prot [3] and compared to ERVEV with ClustalW [23] based on protein sequences (all GenBank and Swiss-Prot accession numbers for the used proteins can be found in Fig. 1).

#### Phylogenetic analysis

For phylogenetic analysis of the ERVEV proteins, ERVEV-specific sequences were aligned to a selection of available corresponding Nairovirus sequences from GenBank by means of the ClustalW algorithm (neighbor-joining method) of commercially available MegAlign software version 9.1.0 (DNAStar, Lasergene), MEGA version 5.05 [22] and *DENDROSCOPE* [12] with a 1,000-fold bootstrap approach.

## Results

Using combined FLAC anchor primers for the 3′ end and 5′ RACE adaptors pyrosequncing determined the full-length sequences of the 3 genome segments of ERVEV in 1 lane of a 4-lane picotiter plate in a pool of 6 different RL-MID-tagged virus libraries in one sequencing run. The ERVEV library yielded 42,025 reads (11,173,117 bases) with 19,455 specific reads (= 46 %) or 5,174,304 bases (average alignment depth (AAD) = 289). All 3 genome segments were covered by 100 %.

Table 1 shows the length of the ERVEV genome segments and the calculated theoretic molecular mass of the encoded viral proteins. The complete genome size is 17,907 bp. Table 2 lists the 5′ and 3′ non-coding sequences of the genome segments of ERVEV. The 5′ NCRs contain 12 conserved nucleotides and the 3′ NCRs 11 conserved nucleotides. The first and the last 9 nucleotides of all ERVEV segments are inverted complements. Table 3 shows the amino acid length of the putative ERVEV proteins RdRP, M-protein, and N in comparison to the corresponding proteins of other Nairoviruses. At a length of 630 amino acids (aa), 1,296 aa, and 3,863 aa, N, M-protein, and RdRP are, respectively, 145 aa longer, 125 aa shorter, and 60 aa shorter than the hitherto biggest known N, the shortest known M-protein, and the shortest known RdRP of all Nairovirus proteins described for HAZV.

**Table 1:** Lengths and encoded putative proteins of ssRNA segments L, M, and S of ERVEV

Segment	Length (bp)	Encoded viral protein	Length (aa)	Mass^a^ (Da)
ERVEV				
Segment L	11,684	RdRP	3,863	440,398
Segment M	4,043	Glycoprotein precursor	1,296	144,604
Segment S	2,180	Nucleocapsid	630	69,870

^a^Calculated theoretic molecular mass

**Table 2:** 5′ and 3′ non-coding regions (NCRs) of ERVEV segments L, M, and S. Conserved bases are indicated in capital letters and inverted complements are underlined

Segment	5′-NCR		3′-NCR	
	Length (bp)	Terminal sequence	Terminal sequence	Length (bp)
ERVEV				
Segment L	38	5′-UCUCAAAGAAAGca…	…acUAUCUUUGAGA-3′	54
Segment M	40	5′-UCUCAAAGAAAGac…	…uaUAUCUUUGAGA-3′	112
Segment S	44	5′-UCUCAAAGAAAGuu…	…acUAUCUUUGAGA-3′	243

**Table 3:** Comparison of the protein sizes of the different Nairoviruses

	CCHFV	HAZV	NSDV	DUGV	KUPV	ERVEV
RdRP	3945aa	3923aa	3991aa	4036aa	4050aa	3863aa
M-protein	1684aa	1421aa	1627aa	1551aa	1549aa	1296aa
Nucleocapsid	482aa	485aa	482aa	483aa	483aa	630aa

### Phylogenetic analysis

Sequence distance analysis for all available Nairovirus protein sequences in comparison to putative CCHFV protein sequences showed a low degree (<49 %) of similarity of the 3 putative ERVEV protein sequences with those of CCHFV (Fig. 1a–c). Neigbor-joining phylogenetic trees for the RdRP, M-protein, and N sequences of all available Nairoviruses rooted to the corresponding sequences of the Orthobunyavirus Bunyamwera virus (BUNV) clearly show that ERVEV represents the most divergent Nairovirus analyzed to date (Fig. 1a–c). Phylogenetic comparison of the putative amino acid sequence of the RdRP revealed only 48 % homology to available RdRP sequences of other Nairoviruses like CCHFV, NSDV, HAZV, KUPV, and DUGV. Among themselves, these Nairoviruses show 63–89 % homology in the RdRP sequences (Fig. 1a). A similar pattern can be seen for the N proteins with ERVEV N showing only 42–44 % identity to other Nairovirus N proteins, whereas the homology among the other Nairoviruses is between 56 and 75 % (Fig. 1c). The homologies between the glycoprotein precursors are less pronounced (Fig. 1b), probably because these surface proteins that directly interact with the adaptive immune system have to face a higher evolutionary pressure compared to the RdRP or the nucleocapsid protein.

**Fig. 2:**
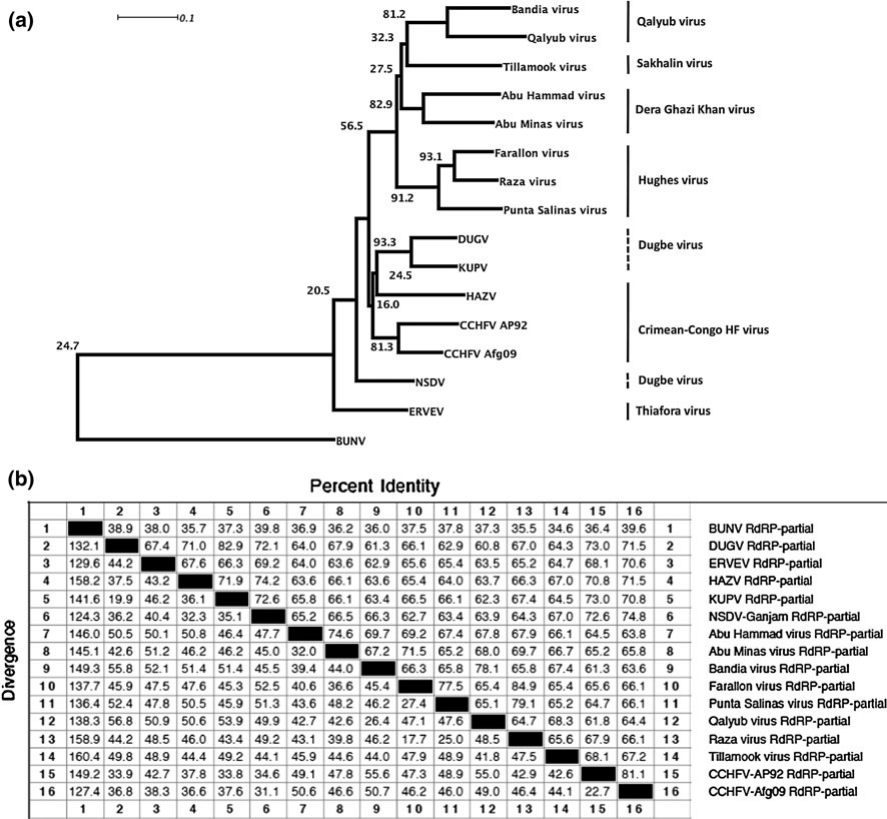
**a** Neighbor-joining phylogenetic tree of a 442-bp fragment (413-bp fragment for Qalyub virus) of the nucleic acid sequence of the RdRP gene of different Nairoviruses by means of MegAlign version 9.1.0 (DNASTAR, Lasergene) with the multiple alignment algorithm ClustalW and a 1,000 fold bootstrap approach. Bootstrap values are given in percent. *DENDROSCOPE* [12] was used to edit the tree. The different Nairovirus serogroups based on the Eight and Ninth Report of the International Committee on Taxonomy of Viruses [17, 19] are indicated. **b** Sequence pair distance of the nucleic acid sequences of a 442-bp fragment (413-bp fragment for Qalyub virus) of the RdRP gene of different Nairoviruses by means of MegAlign version 9.1.0 (DNASTAR, Lasergene) with the multiple alignment algorithm ClustalW

Since there are more partial sequences available for other Nairoviruses than complete genomes, we assembled all available partial sequences of the RdRP into a neighbor-joining phylogenetic tree (Fig. 2a), which again indicates that ERVEV is the most distinct Nairovirus analyzed to date. The sequence pair distance (Fig. 2b) shows only 62.7–70.6 % identity of the 442-bp fragment of ERVEV RdRP compared to other Nairoviruses.

### Comparison of Nairovirus OTU domains

**Fig. 3:**
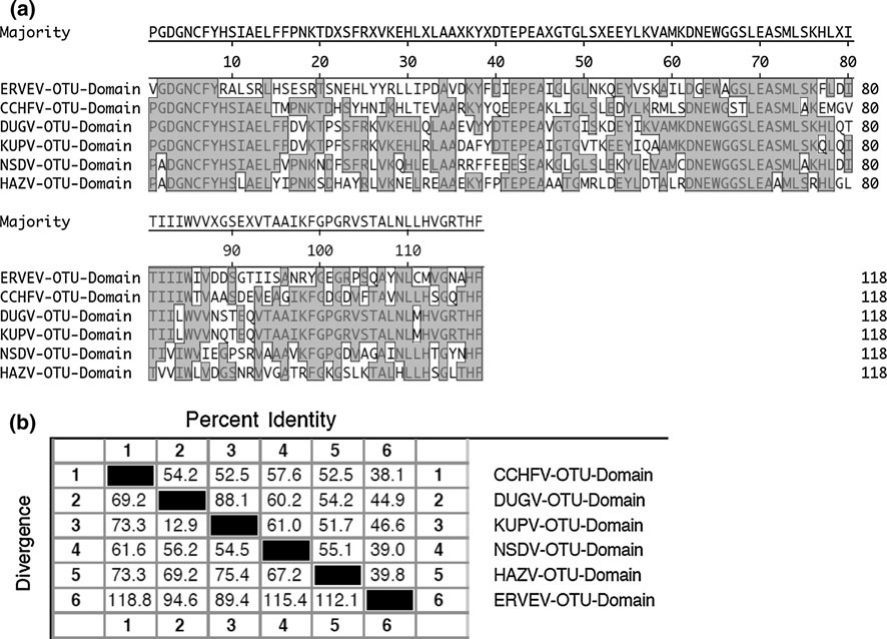
**a** Multiple alignment of protein sequences of the OTU domains of different Nairoviruses by means of MegAlign version 9.1.0 (DNASTAR, Lasergene) with the multiple alignment algorithm ClustalW. Residues that match the consensus exactly are boxed and shaded in *gray*. Positions of the first and last aligned residue in the protein sequence of the respective L proteins: DUGV (35-152), KUPV (35-152), NSDV (35-152), HAZV (35-152), CCHFV (35-152), ERVEV (38-155). **b** Sequence pair distances of the protein sequences of the different Nairovirus OTU domains by means of MegAlign version 9.1.0 (DNASTAR, Lasergene) with the multiple alignment algorithm ClustalW

Innate immune cytokines such as type I interferon (IFN-α/β) and tumor necrosis factor alpha (TNF-α) play fundamental roles in the early response to viral infection. The gene expression of both cytokines depends on the host proteins Ubiquitin (Ub) and interferon-stimulated gene product 15 (ISG15) which reversibly conjugate to proteins via a conserved LRLRGG C-terminal motif. This common structure motif can be targeted by viruses to simultaneously avoid both IFN-α/β and TNF-α effects. Ub and ISG15 are synthesized as inactive precursors that undergo cleavage to expose the LRLRGG sequence required for conjugation to target molecules. The coordinated activities of an enzymatic cascade comprising an activating enzyme (E1), a conjugating enzyme (E2), and a ligase (E3) result in the conjugation of Ub or ISG15 to the ε-amino group of a lysine residue present in the target protein. This conjugation can be reversed by the activity of deconjugation enzymes. In case of Ub, five classes of de-ubiquitinating proteolytic enzymes have been described [18]. One of the most recently identified classes is the ovarian tumor (OTU) domain family of putative cysteine proteases that shows homology to the OTU protein in Drosophila. The OTU domain superfamily comprises more than a hundred proteins found in eukaryotes, bacteria, and viruses [14]. Frias-Staheli et al. [10] showed that the large (L) protein of Nairoviruses, which contains an OTU domain and an RNA polymerase domain, displays a broad deconjugating activity toward ubiquitinated and ISGylated products and consequently inhibits innate immunity pathways which are dependent on Ub and ISG15 to evade the host antiviral response probably by targeting a common biochemical structure in Ub and ISG15. A multiple alignment of the different Nairovirus OTU domains of 118aa length predicted by the Conserved Domains Database (CDD) curated by NCBI [15] can be seen in Fig. 3 a. The sequence pair distance (Fig. 3 b) shows the lowest identity of 38.1 % for the ERVEV OTU domain with that of CCHFV, whereas the other Nairoviruses show identities higher than 52 % with the CCHFV OTU domain.

## Discussion

ERVEV is a Nairovirus described in France, Germany, the Netherlands, and Czech Republic [4]. It is suspected to cause severe headache (thunderclap headache) and intracerebral hemorrhage [24]. The mode of transmission to humans (ticks or mosquitoes) is still unknown. Currently, no standardized testing method for ERVEV exists and only a small partial sequence (442 bp) of the polymerase gene was available. We were able to sequence the complete genome of ERVEV via pyrosequencing with 289-fold coverage. It consists of 3 segments of negative-stranded ssRNA. The encoded proteins show characteristics which mark ERVEV as the most divergent virus among the Nairoviruses analyzed to date. This also includes the OTU domain in the RdRP and the nucleocapsid protein, which is significantly longer (630 aa) than nucleocapsid proteins of other Nairoviruses (on average 483 aa). Homologies of the ERVEV protein sequence in comparison to sequences of other Nairoviruses range from 41.9 to 44.0 % for the nucleocapsid, from 33.5 to 39.1 % for the gylcoprotein precursor, and from 47.9 to 48.3 % for the RdRP. The partial RdRP sequence analysis including 9 more Nairoviruses, although unreliable due to low bootstrap values in parts of the tree, confirms this assessment. In general, Nairoviruses exhibit a high degree of genetic variation, which correlates with the diversity of their tick hosts [11]. An arthropod vector for ERVEV was not identified so far, and tick transmission seems unlikely as no correlation between ERVEV infection and *Borrelia burgdorferi* infections was observed [26]. Taken together, this might indicate that this most divergent Nairovirus ERVEV may be vectored by a very particular arthropod.

The new sequence data can now be used for the development of diagnostic methods especially nucleic acid detection methods or expression cloning and protein purification for serological assays. The developed diagnostic systems could help to identify the natural vector and to clarify the importance of this human pathogen.
